# Coil Embolization of Arc of Buhler Aneurysm Rupture

**DOI:** 10.1155/2020/8855946

**Published:** 2020-12-11

**Authors:** Daniel Yuxuan Ong, Uei Pua

**Affiliations:** Department of Diagnostic Radiology, Tan Tock Seng Hospital, Singapore

## Abstract

The arc of Buhler (AOB) is a rare anatomical variant. Rupture of an AOB aneurysm is exceedingly rare. In this study, we report a case of AOB aneurysm rupture, which was successfully treated by transcatheter coil embolization. A 74-year-old man presented with symptomatic AOB aneurysm rupture. A computed tomography scan and subsequent angiography revealed the aberrant connection between the common hepatic artery and the superior mesenteric artery. A fusiform AOB aneurysm with focus of active bleeding was detected. This was successfully treated through embolization and sacrifice of the AOB. This suggests that conventional embolization with sacrifice of AOB is a feasible and safe approach.

## 1. Introduction

The anastomotic network exists between the celiac artery (CA) and superior mesenteric artery (SMA) and provides crucial blood supply to the foregut and midgut. The two most common anastomoses are via the pancreaticoduodenal arcade and dorsal pancreatic artery. Rarely, a third connection can exist in the form of an arc of Buhler (AOB).

The AOB is an anatomical variant first described by Buhler and Tandler [1]. It is believed to be due to the failure of regression at the ventral segmental anastomosis between the 10th and 13th segmental arteries, leading to the ventral communication between the celiac artery and the SMA. The incidence of AOB is at an estimated less than 3-4%. Most commonly, it appears as a direct anastomotic artery joining the celiac artery and SMA, although anastomotic artery joining the main tributaries of the celiac artery (e.g., splenic or hepatic) and SMA is also known [1]. While patients with AOB are generally asymptomatic, knowledge of this artery is important when contemplating pancreaticoduodenal surgery, hepatic surgery, hepatic embolization, or ablation in this region.

Aneurysm arising from the AOB is a rare entity, and literature surrounding the incidence and management remains sparse. In contrast, patients with AOB aneurysms are commonly symptomatic and can present with nonspecific abdominal pain or rupture [1–4] and are typically diagnosed on CT or angiography.

In this case report, the authors describe a case of AOB aneurysm rupture and how conventional coil embolization with sacrifice of the entire anastomosis is a feasible management option.

## 2. Case Presentation

**Figure 1:**
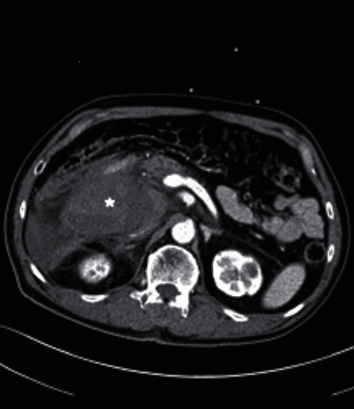
Axial CT image showing a large acute retroperitoneal hematoma (asterisk) centered around the paraduodenal region extending anterior to the right anterior pararenal fascia.

**Figure 2:**
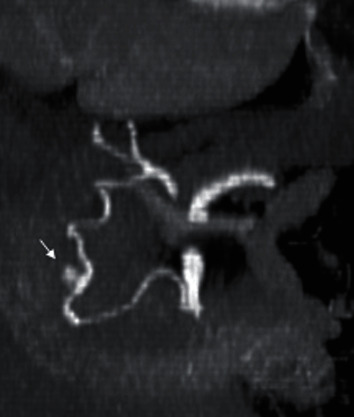
Maximum intensity projection (MIP) image showing a focus of active bleeding (arrow) from the vascular arcade running along the mesenteric side of the duodenal c-loop. This was initially thought to represent the pancreaticoduodenal arcade but was found to be an arc of Buhler during angiography. Fusiform dilatation at the site of the active bleeding could be appreciated.

A 74-year-old man presented with near-syncope and abdominal pain and was then found to be hypotensive (95/62 mmHg) with abdominal guarding. Sonography revealed free fluid in the right hypochondrium which on CT revealed a large retroperitoneal hematoma, predominant in the paraduodenal region (Figure 1). The initial suspicion was active bleeding arising from the pancreaticoduodenal arteries due to the location of hematoma (Figures 1 and 2), and urgent angiography with the view for embolization was performed. During celiac angiography, the gastroduodenal artery (GDA) was not opacified due to reciprocal/competitive hepatopetal flow, but was successfully selected based on landmark using a 2.4 F microcatheter. GDA arteriography showed a complete arcade with no evidence of bleeding. The pancreaticoduodenal arcade was intact as well. In view of the emergent clinical status, the GDA was prophylactically embolized using MVP 3Q and 5Q (Medtronic, Minneapolis, MN) based on the CT finding, with a view for additional retrograde embolization based on superior mesenteric arteriography findings.

**Figure 3:**
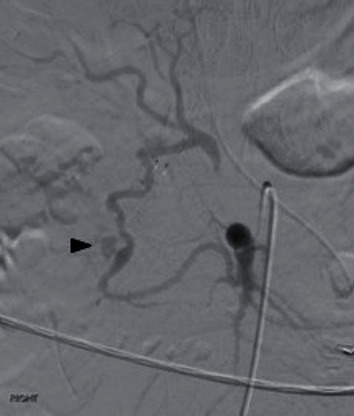
Superior mesenteric arteriography showing the arc of Buhler with a short segment of fusiform dilatation and active extravasation (arrowhead). Hepatofugal flow was seen with brisk opacification of the proper hepatic artery. The cause of which was unknown.

**Figure 4:**
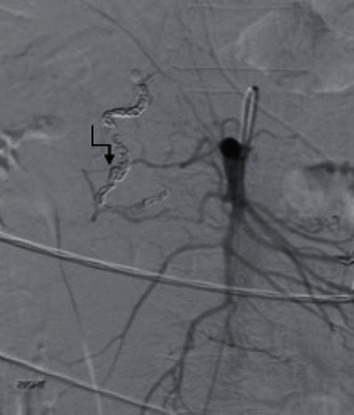
Postembolization angiography demonstrating complete stasis and sacrifice of arc of Buhler (zigzag arrow).

Superior mesenteric arteriography was then performed to detect any retrograde perfusion and bleeding from the pancreaticoduodenal arcade. It was at this time that an aberrant artery communicating between the common hepatic artery and the SMA separate from the pancreaticoduodenal/GDA was detected. The appearance was consistent with an arc of Buhler. In addition to brisk hepatopedal flow, a fusiform aneurysm with a focus of active bleeding was detected (Figure 3). The arc was cannulated from the celiac axis, and the entire arc was sacrificed through embolization with 6-2-4 mm micronester coils (Cook Medical, Bloomington, IN), across the entire aneurysm. Completion angiography from both the celiac artery and SMA showed complete stasis of the arc (Figure 4).

The patient had an uneventful recovery and was discharged well on postoperative day 2 and remained well at 1-month follow-up, with no symptoms of hepatic or bowel ischemia.

## 3. Discussion

In the current literature, unruptured or incidentally discovered AOB aneurysms are treated with prophylactic coil embolization or surgical resection [1–9] (Table 1). Rupture involving an AOB aneurysm is exceedingly rare, with only 2 cases reported. Mohapatra et al. reported the first, where the patient presented with bleeding postsphincterotomy, and the source was found to be from an AOB aneurysm. This was treated with transcatheter embolization [9]. Abe et al. reported the other, in a patient with underlying median arcuate ligament syndrome (MALS) [4]. The MALS was thought to have resulted in hyperdynamic hepatofugal flow with resultant aneurysm formation and rupture. The saccular AOB aneurysm was embolized leaving the AOB intact [4].

In contrast, our case is the first where there is complete sacrifice of the AOB. Theoretically, this technique has several potential risks, such as resultant hepatic or bowel ischemia. The aneurysm of the AOB has a fusiform morphology as opposed to the saccular morphology in the prior publications [4, 9]. The fusiform morphology would require sacrifice of the entire artery as conventional coiling techniques in saccular aneurysm ruptures were not feasible. Angiography showed good collateral flow to the relevant areas, and hence, sacrificing the entire AOB was considered safe. The patient was closely followed up for postoperative hepatic and bowel ischemia and remained well.

In our case, we were uncertain of the cause for the hepatofugal flow through the AOB. Retrospective review of both the CT and angiography reviewed no stenosis of the celiac axis or definite imaging evidence of MALS.

## 4. Conclusion

The above case adds to our understanding of the rare occurrence of AOB aneurysm rupture and that coil embolization sacrificing the entire anastomosis is a feasible treatment option.

## Figures and Tables

**Table 1:** 

Study	Demographics	Arc of Buhler	Management	Outcome
Kugai et al.	59 years old, female	Unruptured	Surgery	Success
Myers et al.	39 years old, female	Unruptured	Surgery	Success
Dubel et al.	54 years old, male	Unruptured	Embolization of parent artery	Success
Jeong et al.	41 years old, male	Unruptured	Embolization of parent artery	Success
Jayia et al.	65 years old, female	Unruptured	Embolization of aneurysm	Success
Sugihara et al.	35 years old, male	Unruptured	Embolization of aneurysm	Success
Mohapatra et al.	53 years old, male	Ruptured	Embolization of parent artery	Success
Abe et al.	60 years old, female	Ruptured	Embolization of aneurysm	Success

Reported arc of Buhler aneurysms and management.

## Data Availability

The data supporting the results of the study has been included in the manuscript.
